# Exploring Correlates of Loss of Control Eating in a Nonclinical Sample

**DOI:** 10.3389/fpsyg.2021.787558

**Published:** 2022-02-11

**Authors:** Eva M. Conceição, Célia S. Moreira, Marta de Lourdes, Sofia Ramalho, Ana Rita Vaz

**Affiliations:** ^1^Psychotherapy and Psychopathology Research Unit – Psychology Research Centre, School of Psychology, University of Minho, Braga, Portugal; ^2^Department of Mathematics and Center of Mathematics (FCUP-CMUP), University of Porto, Porto, Portugal

**Keywords:** self-criticism, loss of control eating, non-clinical sample, eating disorders psychopathology, emotion regulation

## Abstract

**Objective:**

Loss of control (LOC) eating has been directly related to the core aspects of the psychopathology of eating disorders and to different dimensions of emotion and behavior regulation and self-criticism. This study investigates a model representing the interplay between these dimensions to understand LOC eating among a nonclinical sample.

**Methods:**

A total of 341 participants, recruited in a college campus (mean age 23.21, SD = 6.02), completed a set of self-report measures assessing LOC eating, weight suppression, psychopathology of eating disorders, depression, negative urgency, emotion regulation difficulties, and self-criticism. Path analysis modeling tested a hypothesized model with 3 paths for LOC eating as follows: (1) psychopathology of eating disorders; (2) emotion and behavior regulation; and (3) interplay between these paths.

**Results:**

We found goodness-of-fit indexes to our data: χ^2^ = 17.11, df = 10, Comparative Fit Index (CFI) = 0.99, Tucker-Lewis index (TLI) = 0.98, Root Mean Square Error Approximation (RMSEA) = 0.045, Standardized Root Mean Square Residual (SRMR) = 0.041, suggesting that: (1) participants with higher weight suppression showed higher degrees of the psychopathology of eating disorders, which was linked to higher levels of LOC eating; (2) self-criticism was a mediator between emotion regulation and depression/negative urgency; (3) self-criticism was a mediator between emotion regulation and disorder eating, which was significantly associated with LOC eating *via* increased negative urgency.

**Conclusion:**

Our model shows that LOC eating occurs for individuals with the psychopathology of higher eating disorders who experience depressive symptoms and act rashly under distress for their inability to cope adequately with negative feelings of self-devaluation. These findings point to the importance of negative self-evaluations and feelings of inadequacy or worthlessness to understand LOC eating among college students.

## Introduction

Loss of control (LOC) eating is described as the subjective perception of being compelled to eat or unable to resist or stop eating. LOC eating has been considered a core symptom of several eating disorders, including binge-eating disorder, bulimia nervosa, and anorexia nervosa-binge/purge subtype (American Psychiatric Association [APA], 2013). The experience of LOC eating and the consumption of an unambiguously large quantity of food in a discrete period of time are considered the two hallmark features in the definition of binge eating of the DSM-5 (American Psychiatric Association [APA], 2013). However, cumulative evidence suggests that the experience of LOC eating, rather than episode size, is the most salient indicator of the psychopathology of eating disorders and psychological distress in clinical and nonclinical samples of both in the youths (Schlüter et al., 2016; Byrne et al., 2019) and adults (Mond et al., 2010; Fitzsimmons-Craft et al., 2014; Goldschmidt, 2017). Current research also suggests that the degree of LOC eating is associated with the degree of eating-related psychopathology and psychological distress in bariatric surgery samples (Conceição E. M. et al., 2018), adults (Latner et al., 2014), and adolescents (Vannucci and Ohannessian, 2018), in a way that the greater the degree of LOC eating experienced, regardless of the amount of food eaten, the more severe are eating-related symptoms and distress. Although LOC eating is conceptualized as directly related to the core aspects of the psychopathology of eating disorders, particularly the importance and concerns over eating and body weight/shape (Fairburn et al., 2003), other variables have been investigated to explain the relationship between core psychopathology of eating disorders and LOC eating behavior.

### Psychological and Weight-Related Variables Associated With Loss of Control Over Eating

*Weight suppression* represents the difference between the highest lifetime weight and current weight and is considered a transdiagnostic factor in eating disorders (Lowe et al., 2018). Weight suppression has been associated with more shape and weight concerns, higher restraint, and eating disorders severity in individuals with eating disorders (Lowe et al., 2018) and nonclinical undergraduates (Burnette et al., 2018). In the community sample, weight suppression was found to be a predictor of binge-eating behavior and more frequent LOC eating (Van Son et al., 2013).

*Overvaluation over eating, body weight, and body shape* are considered the core-psychopathology of eating disorders, and self-evaluation is overdependent on the control exerted over these dimensions (Fairburn et al., 2003). In the context of eating disorders, self-evaluation is also dependent on the achievement of demanding and self-imposed standards in the control of these dimensions (Shafran et al., 2002; Fairburn et al., 2003). Failure to meet those standards results in self-critical thoughts toward oneself and strengthens the concerns over body shape, weight, and eating (Shafran et al., 2002).

Consistent with this perspective, *self-criticism* has been suggested as a strong predictor of the psychopathology of eating disorders (Fennig et al., 2008) in patients with binge eating disorders (Dunkley and Grilo, 2007) and college students (Porter et al., 2018). Self-criticism also seems to have significant longitudinal relations with fasting, purging, and excessive exercise (Zelkowitz and Cole, 2020). It involves negative self-evaluations and feelings of inadequacy or worthlessness (Gilbert et al., 2004). The relationship between these self-critical feelings and psychopathology of eating disorders may be partially mediated by *depressive symptoms* and low self-esteem (Dunkley and Grilo, 2007), which are reinforced by pursuing and failing to meet high standards on the dimensions of eating, weight, and shape control. Consistently, Feinson and Hornik-Lurie (2016) suggested that binge eating may be a self-soothe strategy from the negative effect triggered by self-critical thoughts that result from failing to meet the self-imposed high/demanding standards (Feinson and Hornik-Lurie, 2016).

Along these lines, a growing body of research suggests that *emotion regulation* also seems to play a role in the link between self-criticism and binge eating/LOC eating. Different studies showed that poor emotion regulation skills are prospectively and concurrently associated with binge eating and LOC eating in adolescents (Goldschmidt et al., 2017), college students (Markey and Vander Wal, 2007), and adults (Bodell et al., 2019), even after controlling for negative affect (Markey and Vander Wal, 2007). Moreover, negative affect seems to increase prior and decreases after binge eating (Berg et al., 2015; Schaefer et al., 2020), and there is evidence that the learned expectancy that eating will alleviate distress seems to increase the risk for binge eating (Fischer et al., 2013). These findings are consistent with the affect regulation model of binge eating (Haedt-Matt and Keel, 2011; Leehr et al., 2015) arguing that binge eating may serve as an emotion regulation process in individuals lacking adaptive strategies and show that this relationship may not be exclusive to individuals with eating disorders.

Finally, the tendency to act impulsively under situations of negative emotionality (*negative urgency*) may provide additional insight on the behavioral relation between emotion regulation difficulties and LOC eating. Negative urgency is considered the facet of impulsivity mostly associated with binge eating (Kelly et al., 2014) and seems to be significantly associated with both LOC eating and the ingestion of large amounts of food (Racine et al., 2015). Research has consistently shown that higher negative urgency scores are prospectively (Emery et al., 2013; Fischer et al., 2013) and cross-sectionally (Kelly et al., 2014; Aloi et al., 2020) associated with binge-eating severity in clinical or community samples. Additionally, greater negative urgency combined with greater negative emotionality is associated with the psychopathology of eating disorders in women with and without bulimic disorders (Conceição E. et al., 2018; Magel and von Ranson, 2021). Considering this research, individuals with poorer emotion regulation strategies, particularly those who tend to act rashly when experiencing negative emotions, would tend to engage more in LOC eating.

### The Interplay Between Weight-Related and Psychological Aspects to Understand Loss of Control Over Eating

Despite the extensive body of literature regarding each of these factors (weight suppression, psychopathology of eating disorders, self-criticism, depressive symptoms, and negative urgency), to the best of our knowledge, no study investigated how these aspects interact with each other to explain LOC eating. This research is particularly scarce in nonclinical samples despite the growing evidence that LOC eating in this population is highly prevalent and associated with the psychopathology of eating disorders, psychological distress, and body mass index (BMI) (Mustelin et al., 2017). This research can have important implications for identifying the mechanisms that link psychopathology of eating disorders and emotion regulation under distress, and to inform our understanding of LOC eating in nonclinical conditions.

**FIGURE 1:**
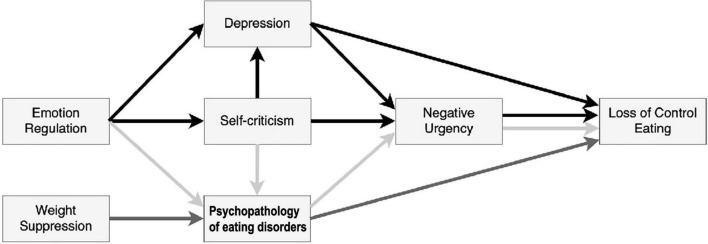
Hypothesized model depicting the interplay between disordered eating-related variables and other psychological variables to explain loss of control (LOC) eating. 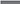
Path 1 – Psychopathology of eating disorders and LOC eating; 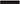
Path 2 – emotion and behavior regulation under distress; and 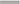
Path 3 – interplay between Path 1 and Path 2.

Taking into consideration the data previously described, we proposed the model of LOC eating presented in Figure 1. In this model, we hypothesized three paths to understand LOC eating in a nonclinical sample:

*Path 1 –Psychopathology of eating disorders and LOC eating*: increased weight suppression (greater variations in the weight of individuals) would be associated with eating disorders (eating and weight/shape concerns), which would be positively and significantly associated with higher LOC eating;

*Path 2 – Emotion and behavior regulation under distress*: emotion regulation difficulties would be associated with greater self-criticism (thoughts of self-inadequacy and self-failure), leading to more depressive symptoms, which would increase LOC eating directly or *via* greater levels of negative urgency.

*Path 3 – Interplay between Path 1 and Path 2*: self-criticism (feelings of failure/negative self-evaluation) and emotion regulation difficulties (to deal with such negative experiences of the self) would be associated with LOC eating *via* increased psychopathology of eating disorders. Psychopathology of eating disorders would be associated with LOC eating *via* increased levels of negative urgency.

## Materials and Methods

### Participants and Procedure

Recruitment was conducted in a university campus in the north of Portugal from January to April 2018. All students from the university institution received an invitation to participate in this study in their institutional email. Survey invitation letters provided a link to the questionnaires *via Google Docs* software. Advertising on Facebook was also used to disseminate the invitation. Participants not fluent in written Portuguese were excluded. All participants were informed about the aims, as well as the voluntary and confidential nature of the study. Participants agreed on an informed consent form and filled out a set of questionnaires that took approximately 20 min. To enhance the response rate, a raffle draw of a €20 voucher to use in a main retail chain store following survey completion was run with accepting participants. The institutional review board from the university involved approved this study.

### Measures

*Sociodemographic and anthropometric questionnaire:* a measure that included items self-reported about sociodemographic (age, gender, educational level, and professional status) and anthropometric information (height, current, highest, and lowest weight in adulthood). Weight suppression relative to current weight was computed as [current weight suppression = highest weight - current weight]; weight suppression relative to lowest weight was computed as [lifetime weight suppression = highest weight - lowest weight].

*The Loss of Control over Eating Scale (LOCES)* (Latner et al., 2014): A 24-item self-report measure intended to measure the degree of LOC eating. Responses ranged from 1 (“never”) to 5 (“always”) focuses in the past 28 days, which were averaged to generate a total score. The items are organized into three factors: (1) the behavioral aspects of LOC eating (e.g., “I kept eating although I was no longer hungry”), (2) the cognitive/dissociative aspects of LOC eating (e.g., “I could not concentrate on anything other than eating”), and (3) the positive/euphoric aspects of LOC eating (e.g., “While eating, I felt a sense of relief or release”). Higher results indicate higher LOC eating. The Cronbach’s α obtained in the present investigation was 0.95 for the total score. Validation of the Portuguese version of this measure is currently ongoing and being prepared for publication. Translation and back-translation and a pilot test with the target audience were conducted and followed by larger recruitment with clinical and community samples.

*Eating Disorder-15 (ED-15)* (Tatham et al., 2015; Rodrigues et al., 2019): This measure includes a 10-item measure answered in a 7-point Likert scale from 0 (“Not at all”) to 6 (“All the time”) that assesses behaviors, attitudes, and feelings associated with eating disorders. This questionnaire generates two subscale scores (weight and body shape concern, and eating concern) and a combined global severity score. Higher scores indicate psychopathology of greater eating disorders. In this study, only the total score was used (α = 0.94 for our sample).

*Depression, Anxiety and Stress Scales (DASS-21)* (Lovibond and Lovibond, 1995; Pais-Ribeiro et al., 2004): A self-report measure that assesses the magnitude of the following three negative emotional states: depression, anxiety, and stress. For this study, only the depression scale of this questionnaire was used. The corresponding 7 items are answered in a 4-point Likert scale from 0 (“Did not apply to me at all”) to 3 (“Applied to me very much or most of the time”) during the previous week. Higher scores express greater distress. Cronbach’s α for our sample was 0.96 for the depression subscale.

*Urgency, Premeditation, Perseverance, and Sensation Seeking Scales – Negative urgency (UPPS-NU)* (Whiteside et al., 2005; Conceição et al., 2020): The subscale of negative urgency of the UPPS was used. Negative urgency is the tendency of an individual to act rashly under situations of negative emotions. The 12-item scale is answered on a 4-point Likert scale from 1 (“Completely agree”) to 4 (“Completely disagree”) assess. Higher scores indicate greater negative urgency. Cronbach’s α for our sample was 0.91 for the total score.

*Difficulties in Emotion Regulation Scale (DERS)* (Gratz and Roemer, 2004; Veloso et al., 2011): A 36-item self-report measure that assesses clinically relevant difficulties in emotion regulation through a total score and six dimensions. For this study, only the total score of this questionnaire was used. Responses ranged from 1 (“Almost never”) to 5 (“Almost always”). Higher scores indicate increased difficulties with emotion regulation. Cronbach’s α for our sample was 0.95 for the total score.

*Forms of Self-Criticizing and Reassuring Scale (FSCRS)* (Gilbert et al., 2004; Castilho and Gouveia, 2011): A 22-item self-report measure that assesses how people tend to self-criticize and self-reassure themselves, toward failure and error situations. Items are rated on a five-point Likert scale from 0 (“Anything like me”) to 4 (“Extremely like me”) and generate three subscales. For this study, only the self-criticism subscales were used as a single variable corresponding to the sum of the inadequate self and hated self-subscales. Higher scores indicate greater self-criticism feelings/thoughts. Cronbach’s α for our sample was 0.94 for the computed total score.

### Statistical Analyses

Spearman’s rho coefficients were conducted to investigate the correlation between LOC eating and the other variables under study. This analysis was conducted using the IBM^®^ SPSS^®^ Statistics 25.0 (SPSS Inc., Chicago, IL, United States) for windows.

The path analyses were conducted using the R statistical environment (RStudio, version 3.6.2, R Development Core Team, 2018), through the package “lavaan” (Rosseel, 2012). The significance level was set at α = 0.05. Structural equation modeling (SEM) tools were used to assess the validity of the path model by fitting it to the observed data. Several fit indices were used to assess the model fit: chi-square statistic, degrees of freedom (df), the Comparative Fit Index (CFI), the Tucker-Lewis index (TLI), the Root Mean Square Error Approximation (RMSEA), and the Standardized Root Mean Square Residual (SRMR). Goodness-of-fit model is indicated by a nonsignificant chi-square test, TLI and CFI greater than 0.95, RMSEA smaller than 0.06, and SRMR smaller than 0.08 (Hu and Bentler, 1999). The bootstrapping method was selected to compute SEs of the parameter estimates. All continuous variables were standardized and centered. The full information maximum likelihood (FIML) method was selected to deal with missing data. The hypothesized model was tested and the nonsignificant paths between variables were dropped to improve the model until we reached the final model.

## Results

A total of 341 participants aged between 18 and 59 years (*M* = 23.21, SD = 6.02) responded to our survey. Participants included 246 (72.1%) women and 95 (27.9%) men. Within the sample, 58 (17%) participants attended their 1st year of college, 78 (22.9%) attended their final (5th) year, 173 (50.7%) spread through the 2nd and 4th year, and 32 (9.4%) were doctoral or postgraduate students. The majority of participants, 285 (83.6%), were students, 45 (13.2%) were student-workers, and 11 (3.2%) identified as other. Mean current, highest, and lowest BMI was 22.91 (SD = 4.40), 24.47 (SD = 4.98), and 20.75 (SD = 3.61) kg/m^2^, respectively. Mean current weight suppression and mean lifetime weight suppression was 4.33 (SD = 4.33) and 10.28 (SD = 7.95), respectively.

Table 1 presents the correlations between LOC eating and the other psychological variable under study. Although correlations between the variables under study were all statistically significant, LOC eating showed particularly strong correlations (> 0.4) with the psychopathology of eating disorders (ED-15), and negative urgency (UPPS-NU). Interestingly, psychopathology of eating disorders was strongly (> 0.4) correlated only with self-criticism (FSCRS). Of note, lifetime weight suppression was more strongly associated with psychopathology of eating disorders and LOC eating than weight suppression relative to current weight. Self-criticism was further strongly correlated with difficulties in emotion regulation, negative urgency, and depression.

**TABLE 1:** Correlation between variables under study.

		1	2	3	4	5	6	7	8
1	Loss of control eating (LOCES)	–							
2	Eating disorder psychopathology (ED-15)	**0.52** ***	–						
3	Current weight suppression	0.01	0.12*	–					
4	Life-time weight suppression	0.21***	0.28***	**0.53** ***	–				
5	Self-criticism (FSCRS)	0.36***	**0.54** ***	−0.02	0.14*	–			
6	Negative urgency (UPPS-NU)	**0.41** ***	0.32***	0.01	0.19**	**0.45** ***	–		
7	Depression (DASS)	0.37***	0.35***	−0.08	0.07	**0.58** ***	**0.40** ***	–	
8	Emotion regulation (DERS)	0.25***	0.34***	−0.04	0.11	**0.55** ***	0.32***	0.35***	–

*LOCES – The Loss of Control over Eating Scale; ED-15 – Eating Disorder-15; Depression, FSCRS – Forms of Self-Criticizing and Reassuring Scale, sum of the inadequate-self and hated self; UPPS-NU – Urgency, Premeditation, Perseverance, and Sensation Seeking scales; DASS – Depression, Anxiety and Stress Scales; DERS – Difficulties in Emotion Regulation Scale.*

* p < 0.05, ** p < 0.01, *** p < 0.001. Strong correlations (> 0.4) are highlighted in bold.

**FIGURE 2:**
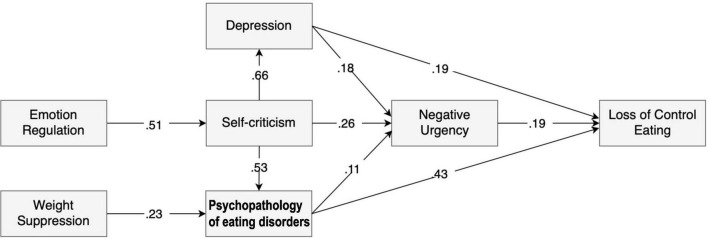
LOC over eating: structural equation model depicting the interplay between disordered eating-related variables and other psychological variables. All paths represented between each variable are significant with *p* < 0.05. Path 1 – Weight suppression → Psychopathology of eating disorders → Loss of control eating. Path 2 – Emotion regulation → Self-criticism → Depression → Negative urgency → Loss of control eating. Path 3 – Self-criticism → Psychopathology of eating disorders → Negative urgency → Loss of control eating.

Figure 2 depicts the final model that reached goodness-of-fit indexes and depicts the complex web of interactions between the variables under study. The main parameter estimates are summarized in Table 2, and the mediation estimates are presented as Supplementary Material (Supplementary Table 1). The relationships between the main variables in this study were specified based on theoretical findings (hypothesized model in Figure 1) and on the correlation matrix presented in Table 1. The model depicted in Figure 2 exhibited very goodness-of-fit indexes to the data with the following goodness-of-fit measures: chi-square = 17.11, df = 10, *p*-value = 0.72, CFI = 0.99, TLI = 0.98, RMSEA = 0.045 with 90% CI = [0.000, 0.082], and SRMR = 0.041.

**TABLE 2:** Parameter estimation of the final model outlined in Figure 2.

Parameters	Estimate	SE	*p*-value	95% bootstrap CI
**Paths (regressions)**				
Emotional regulation → Self-criticism	0.51	0.065	< 0.001	[0.384, 0.634]
Self-criticism → Depression	0.66	0.041	< 0.001	[0.581, 0.744]
Self-criticism → Negative urgency	0.26	0.072	< 0.001	[0.114, 0.396]
Depression → Negative urgency	0.18	0.070	0.011	[0.049, 0.317]
Disordered eating → Negative urgency	0.11	0.057	0.048	[0.010, 0.226]
Weight suppression → Disordered eating	0.23	0.050	< 0.001	[0.138, 0.336]
Self-criticism → Disordered eating	0.53	0.051	< 0.001	[0.425, 0.631]
Depression → Loss of control eating	0.19	0.070	0.008	[0.045, 0.312]
Negative urgency →Loss of control eating	0.19	0.054	0.001	[0.085, 0.305]
Disordered eating → Loss of control eating	0.43	0.057	< 0.001	[0.324, 0.546]
**Covariances**				
Emotional regulation ∼∼ Weight suppression	0.09	0.000	NA	NA
**Variances**				
Self-criticism	0.74	0.090	< 0.001	[0.580, 0.928]
Depression	0.56	0.049	< 0.001	[0.458, 0.654]
Negative urgency	0.78	0.071	< 0.001	[0.641, 0.916]
Disordered eating	0.64	0.054	< 0.001	[0.538, 0.752]
Loss of control eating	0.60	0.052	< 0.001	[0.491, 0.695]
Emotional regulation	1.00	0.000	NA	NA
Weight suppression	1.00	0.000	NA	NA

This model brings support to the hypothesized paths to understand LOC eating. *Path 1 – Psychopathology of eating disorders and LOC eating*, entailing the psychopathology of eating disorders in which participants with higher weight suppression had higher levels of eating disordered disorders psychopathology, which was strongly associated with LOC eating. *Path 2 – Emotion and behavior regulation under distress*, entailing mechanisms of emotion and behavior regulation in relation to depressive symptoms and feelings of self-criticism. Emotion regulation was only indirectly associated with LOC eating through the mediation of self-criticism, which, in turn, associates with LOC eating through the mediation of depressive symptoms and negative urgency. The hypothesized direct paths between emotion regulation and depression/negative urgency were not significant and thus were removed from the final model. Additionally, negative urgency also mediated the relationship between depression and LOC eating. The hypothesized direct path between depression and LOC eating was also significant. Finally, we hypothesized *Path 3 – Interplay between Path 1 and Path 2*, entailing the interplay between the psychopathology of eating disorders (*Path 1*) and the emotion and behavior regulation (*Path 2*). We found that emotion regulation (*Path 2*) was only indirectly associated with LOC eating through the mediation of self-criticism (*Path 2*), which, in turn, is associated with LOC eating through the mediation of psychopathology of eating disorders (*Path 1*). The hypothesized direct paths between emotion regulation and psychopathology of eating disorders were tested but found to be nonsignificant in the model. Additionally, the relationship between psychopathology of eating disorders (*Path 1*) and LOC eating was also partially mediated by negative urgency (*Path 2*).

## Discussion

This study sought to investigate how weight suppression, psychopathology of eating disorders, self-criticism, depression, and emotion and behavior regulation deficits interact to understand LOC eating among a nonclinical sample of college students.

Overall, our model shows that emotion dysregulation is associated with self-criticism, which, in turn, associates with LOC eating through the mediation of psychopathology of eating disorders, negative urgency, and depression. Moreover, weight suppression is associated with the psychopathology of eating disorders, which, in turn, is associated with LOC eating directly and indirectly *via* the mediation of negative urgency.

Specifically, we found support for the hypothesized paths and most of the associations between the variables under study. Regarding *Path 1 – Psychopathology of eating disorders and LOC eating*, our data suggest that LOC eating tends to occur in individuals who score higher on levels of weight/shape and eating concerns. Psychopathology of eating disorders was the variable most strongly associated with LOC eating, which sets the context for this eating disordered behavior. In line with past research, weight suppression was associated with LOC eating (Van Son et al., 2013), but only through the mediation effect of the psychopathology of eating disorders.

Interestingly, our data show that lifetime weight suppression and not current weight suppression had a role in our model. This may suggest that the past experience of weight variation might be a stronger trigger for the development of psychopathology of eating disorders (specifically, weight, shape, and eating concerns), than current weight suppression. Contrary to our findings, other research with clinical samples showed that current weight suppression (Lowe et al., 2018) was strongly associated with eating disorder-related psychopathology and behaviors. In fact, a higher weight suppression may be associated with a stronger bio-behavioral vulnerability (Lowe, 2011), which can contribute to the maintenance of eating and shape concerns, fear of weight gain, and ED behaviors (Bodell and Keel, 2016; Lowe et al., 2018). A possible explanation may rely on disruption in physiological processes in weight-suppressed individuals (e.g., reduced leptin or increased ghrelin levels), which may increase the drive for food consumption and vulnerability to bulimic episodes (Keel et al., 2019). It is possible that the apparently conflicting results reported with our findings are due to the nonclinical nature of our sample and that in this sample, current weight suppression is not clinically severe to induce this biological effect on eating.

Concerning the hypothesized *Path 2 – Emotion and behavior regulation under distress*, our model highlighted the central role of self-criticism as a strong mediator between emotion regulation difficulties and depression/negative urgency, which, in turn, are related to LOC eating. These findings suggest that difficulties dealing with feelings of self-inadequacy or depressive symptoms lead to LOC eating *via* a higher tendency to act rashly under these negative situations. The lack of appropriate emotion regulation skills to deal with thoughts of self-inadequacy and self-failure (self-criticism) results in increased depressive symptoms, which, in turn, lead to LOC eating partially *via* negative urgency. Consistent with past studies, these findings suggest that LOC eating occurs in individuals with greater emotion regulation difficulties (Leehr et al., 2015) who tend to be highly critical about themselves (Feinson and Hornik-Lurie, 2016), feeling more depressed (Dunkley and Grilo, 2007), or acting rashly under negative emotions.

More important to understand LOC eating is *Path 3 – Interaction between Path 1 and Path 2.* Our data show evidence for an interplay between the variables associated with the psychopathology of eating disorders (Path 1) and difficulties in emotion and behavior regulation under negative emotions. Specifically, self-criticism showed to be highly correlated with the psychopathology of eating disorders and to be a strong mediator between emotion regulation and psychopathology of eating disorders, which is associated with LOC eating. These findings highlight the central role of feelings of self-inadequacy and self-failure in the psychopathology of eating disorders and LOC eating. Interestingly, the hypothesized direct paths between emotion regulation and psychopathology of eating disorders, emotion regulation and depression, and emotion regulation and negative urgency were not significant. These data support the argument that, in the context of the psychopathology of eating disorders leading to LOC eating, appropriate emotion regulation skills are needed to specifically deal with self-critic feelings regarding eating, weight, and body shape. Supporting these findings, past research suggests that self-criticism is related to eating disorder symptoms beyond and independently of depression or self-esteem (Dunkley and Grilo, 2007; Porter et al., 2018), probably through a cognitive-personality vulnerability that maintains the eating disorder core-psychopathology (Dunkley and Grilo, 2007). Feinson and Hornik-Lurie (2016) investigated self-criticism, depression, and anxiety and found self-criticism to be the only significant contributor to binge eating severity (Feinson and Hornik-Lurie, 2016). Negative feelings regarding the self may lead to engaging in maladaptive eating (e.g., LOC eating) or weight control behaviors to compensate for the perceived inadequacies or to lessen the negative effect generated from such feelings (Fairburn et al., 2003; Porter et al., 2018). Consistent with the emotion regulation model of binge eating (Leehr et al., 2015), individuals lacking adaptive strategies to cope with negative emotions and feelings of inadequacy resulting from their critical appraisal of the self (triggers) may engage in problematic eating behaviors such as LOC eating (maladaptive emotion regulation behavior) to downregulate such negative states.

Finally, negative urgency served as a partial mediator between psychopathology of eating disorders and LOC eating. This suggests that part of the link between the psychopathology of eating disorders and LOC eating is explained by the tendency to act rashly under negative emotions. Our data provide further evidence for the argument that negative urgency was associated with binge eating above and beyond the influence of attitudes of eating disorders and depressive symptoms (Kelly et al., 2014).

These findings should be read in light of the cross-sectional design of this study. We cannot conclude about causality between these variables neither we can extrapolate these findings for what happens momentarily before or after a LOC eating episode. For instance, recent research with individuals with binge-eating disorder using Ecological Momentary Assessment (EMA) showed that levels of negative affect increase prior to and decreased after binge-eating episodes, suggesting that binge eating may function to alleviate unpleasant emotional experiences (Schaefer et al., 2020). Our data do not allow for such a conclusion but our model fits these EMA findings by depicting a close association between depressive symptom/negative urgency and LOC eating. Future research using EMA technologies to investigate the real-time associations between these variables would certainly expand our knowledge on the cross talk between these measures and how they operate to understand LOC eating.

Future studies should also test this model in clinical samples across the eating disorders and weight spectrum. Although we provided evidence for the interplay between these variables in a nonclinical sample, these conclusions should not be generalized to other samples. For instance, within individuals with eating disorders, emotion regulation difficulties could play a more central role with a more direct link with negative urgency, depression, and eating disorders psychopathology. In eating disorders, emotion regulation difficulties have been shown to improve with eating-disorders treatment (Mallorquí-Bagué et al., 2018), and differentially associated with more restrictive/compulsive eating symptomatology (Monell et al., 2018). Moreover, recent research using an EMA design shows that self-criticism is a potent momentary predictor of cognitions and behaviors of eating disorders in individuals with eating disorders (Mason et al., 2021). In contrast, self-criticism and the hypothesized *Path 1* might not be as salient in the population with overweight/obesity without eating disorders. Current research shows that the experience of LOC eating can appear in association with disordered eating behaviors that are not strongly linked to eating disorders psychopathology (Conceição et al., 2015; Conceição E. M. et al., 2018). Individuals with overweight/obesity may resort to food when under negative emotions or when feeling depressed for its immediate rewarding properties (Lee and Dixon, 2017). Therefore, emotion regulation, depressive symptoms, and negative urgency might place a more central role in explaining LOC eating than self-criticism and eating disorders’ psychopathology among this population.

Finally, our data were collected using self-report measures assessing the perceived experience of LOC eating. Self-report measures are known to overreport LOC eating behaviors (Everett et al., 2021). Although we are not assessing LOC behaviors *per se*, but rather the subjective perception of LOC while eating, it is unclear whether similar findings would apply to LOC eating as assessed by a clinical interview.

Overall, our model shows that LOC eating occurs for individuals with higher eating disorders psychopathology who experience depressive symptoms and act rashly under distress for their inability to cope adequately with negative feelings of self-devaluation. Our results also highlight the central role of self-criticism as a mediator between emotion regulation and psychopathology of eating disorders, depression, and negative urgency in eating. These findings point to the importance of the negative self-evaluations and feelings of inadequacy or worthlessness among college students to understand LOC eating.

## Data Availability Statement

The raw data supporting the conclusions of this article will be made available by the authors, without undue reservation.

## Ethics Statement

The studies involving human participants were reviewed and approved by Ethics Committee for Research in Social and Human Sciences (CEICSH). The patients/participants provided their written informed consent to participate in this study.

## Author Contributions

EC contributed to the planning and design of the study, data analysis and interpretation, took primary responsibility for the manuscript, including reviewing relevant literature, and drafting the manuscript for publication. CM was responsible for data analysis and reporting and interpretation of the results. ML and SR were responsible for data collection, participant recruiting, and contributed to its analysis and interpretation. AV contributed to the design and planning of the study and to its analysis and interpretation. All authors assisted with the literature review and editing of the manuscript and read and approved the final manuscript.

## Conflict of Interest

The authors declare that the research was conducted in the absence of any commercial or financial relationships that could be construed as a potential conflict of interest.

## Publisher’s Note

All claims expressed in this article are solely those of the authors and do not necessarily represent those of their affiliated organizations, or those of the publisher, the editors and the reviewers. Any product that may be evaluated in this article, or claim that may be made by its manufacturer, is not guaranteed or endorsed by the publisher.
