# Supplementary material for: Exploring Correlates of Loss of Control Eating in a Nonclinical Sample
Source: Front Psychol. 2022 Feb 11;12:787558. doi: 10.3389/fpsyg.2021.787558 (PMC8874330; doi:10.3389/fpsyg.2021.787558)
Supplement: Supplementary file 1 [file Table_1.docx]

Supplementary Table 1S: Estimates for all the mediator effects in Figure 2.

| Mediator | Simple path | Indirect effect | | Direct effect | | Total effect |
| --- | --- | --- | --- | --- | --- | --- |
|  |  | **Est.** | **95% bootstrap CI** | **Est.** | **95% bootstrap CI** | **Est.** |
| Depression | Self-criticism 🡪 LOC | .18 | [.07, .30] | .11 | [-.04, .23] | .28 |
| Self-criticism | Emotion regulation 🡪 LOC | .19 | [.13, .25] | .04 | [-.10, .19] | .23 |
| (ED) Eating Disorders’ psychopathology | Weight suppression 🡪 LOC | .16 | [.08, .25] | .07 | [-.05, .21] | .23 |
|  | Self-criticism 🡪 LOC | .28 | [.18, .39] | .11 | [-.02, .25] | .39 |
| Negative urgency | Depression 🡪 LOC | .11 | [.05, .17] | .32 | [.19, .45] | .43 |
|  | Self-criticism 🡪 LOC | .11 | [.05, .18] | .11 | [-.04, .23] | .22 |
|  | ED 🡪 LOC | .08 | [.04, .13] | .49 | [.38, .61] | .57 |

*Note*. Abbreviations: Est. – Estimate; LOC – Loss of control eating. The significance was assessed through the 95% bootstrap confidence interval: intervals that do not include zero indicate significant values. The total effect is the sum of the direct and indirect effects. The path Self-criticism 🡪 LOC was assessed in a multiple mediator model (with both depression and negative urgency).
